# Infant stool color card screening helps reduce the hospitalization rate and mortality of biliary atresia: A 14-year nationwide cohort study in Taiwan: Erratum Chronic kidney disease is associated with upper tract urothelial carcinoma: A nationwide population-based cohort study in Taiwan: Erratum

**DOI:** 10.1097/MD.0000000000005673

**Published:** 2016-12-09

In the articles “Infant stool color card screening helps reduce the hospitalization rate and mortality of biliary atresia: A 14-year nationwide cohort study in Taiwan”^[1]^ and “Chronic kidney disease is associated with upper tract urothelial carcinoma: A nationwide population-based cohort study in Taiwan”,^[2]^ which appeared in Volume 95, Issue 12 and Volume 95, Issue 14, respectively, of *Medicine*, a university affiliation was originally given incorrectly. Dr. Chen's affiliation in both articles should have read “Department of Pediatrics (SC-CC), School of Medicine, College of Medicine, Taipei Medical University, Taipei City, Taiwan”.
